# Validity of parental work information on the birth certificate

**DOI:** 10.1186/1471-2458-8-95

**Published:** 2008-03-25

**Authors:** Jean D Brender, Lucina Suarez, Peter H Langlois

**Affiliations:** 1 Department of Epidemiology and Biostatistics, Texas A&M Health Science Center School of Rural Public Health, 201 SRPH Administration Building, College Station, Texas, 77843-1266, USA; 2 Epidemiology and Disease Surveillance Unit, Department of State Health Services, 1100 West 49th Street, Austin, Texas, 78756, USA; 3 Birth Defects Epidemiology and Surveillance Branch, Department of State Health Services, 1100 West 49thStreet, Austin, Texas, 78756, USA

## Abstract

**Background:**

In the most recent revision (2003) of the U.S. standard certificate of live births, the National Center for Health Statistics recommended that all states collect maternal and paternal usual occupation. Because such information might be useful in the surveillance of job-related risk areas, we assessed the quality of parental work information on the U.S. birth certificate.

**Methods:**

Occupational histories obtained from maternal interviews with Texas (USA) participants in the National Birth Defects Prevention Study were linked to and compared with parental work information on birth certificates. With occupational information from interviews serving as the gold standard, we assessed the quality of occupational information on the birth certificate with measures of sensitivity, specificity, and the kappa statistic.

**Results:**

Of the 649 births available for study, parental occupation agreed between the birth certificate and interview for 77% of mothers and 63% of fathers with similar agreement by case-control status. Among occupations and industries with 10 or more workers by interview, sensitivity of the birth certificate information ranged from 35% to 100% for occupational groups and 55% to 100% for industrial sectors. Specificities of occupations/industries studied ranged from 93 to 100%. Kappa statistics for maternal occupations (0.76 to 0.90) and industries (0.59 to 0.94) were higher than those for paternal occupations (0.48 to 0.92) and industries (0.47 to 0.89). Mothers were frequently misclassified as homemakers or otherwise unemployed while the paternal information was often missing altogether on the birth certificate. Women who worked as health diagnosing and treating practitioners were the least likely (0%) and women in food preparation or serving occupations were the most likely (65%) to be misclassified as not employed on the birth certificate. Among fathers, the proportion of missing occupations was the lowest for occupations in business or financial operations (0%) and highest for occupations in food preparation and serving (30%).

**Conclusion:**

Sensitivity of occupation/industry information on birth certificates varies although the specificity of such information may exceed 95%. Quality of this information also varies by maternal and paternal occupation with misclassification as homemaker a limiting factor among maternal and missing information a limiting factor among paternal work information.

## Background

As part of the National Occupational Research Agenda (NORA) for reproductive health research, the U.S. National Institute for Occupational Safety and Health (NIOSH) recommended increased surveillance activities including the evaluation of exposure data from existing surveillance systems and expansion of birth defects surveillance systems [1]. Numbers of birth defects registries have increased in the past 20 years with over 40 U.S. states and territories having birth defects surveillance systems in place or in the planning phase in 2002 [2]. The establishment of such registries provides opportunities to monitor potential occupational hazards through linkage of birth defect data with occupational information.

In the most recent revision (2003) of the U.S. standard certificate of live births, the National Center for Health Statistics (NCHS) recommended the collection of maternal and paternal usual occupation (worked during the last year) if states could fund the collection and coding of these data [3]. Such information might be useful in studying job-related risk areas. Linkage of birth defects registry cases with their respective birth certificates provides a mechanism to conduct surveillance for reproductive hazards in the workplace. A number of states have collected parental occupation on birth certificates since the 1980s [4]. A survey conducted by Krieger et al. of U.S. health department vital statistics offices revealed that 50% collected data on mother's occupation and 48% on father's occupation [5]. However, the collection of occupation during the past year, usual occupation, or the most recent occupation instead of jobs during the first trimester (maternal exposures) or during the periconceptional period (paternal exposures including take-home exposures) could lead to misclassification of work exposures during the period of greatest vulnerability for morphogenesis of birth defects. Several states have evaluated this potential misclassification including New York State [6,7] and California [8]. The New York State studies were restricted to maternal occupation and found over-reporting of "housewife" on the birth certificate based on work histories obtained from mailed questionnaires. In these studies, questionnaires were mailed one to six years after birth of the index child. The quality of both maternal and paternal occupation was evaluated in a sample of Santa Clara County, California birth certificates; approximately 29% of the mothers' and 20% of the fathers' occupations were misclassified on the certificate based on work information obtained through telephone interviews conducted 2.9 to 6.7 years after birth of the children. In the two more recent studies [7,8], investigators used the U.S. Bureau of the Census 1980 index of industry and occupation to classify parental work information [9], coding systems that have been replaced by the North American Industrial Classification System (NAICS) [10] and the Standard Occupational Classification System (SOC) [11]. Investigators of previous studies concluded that misclassification of parental occupation on the birth certificate would most likely be nondifferential [7] with respect to malformed and normal comparison births and produce measures of association closer to the null (no effect) [8]. While each of these studies examined the degree of agreement between occupational information on the birth certificate and a "gold standard" (information from mail or telephone questionnaires), neither study compared the distribution of occupations and industries with respect to missing values on the birth certificate. Two studies [6,7] provided analyses of the degree of misclassification as homemaker or employment status across maternal occupations on the birth certificate, but the categories were very broad in one study [7] and misclassification across industrial sectors was not examined in the other study [6].

In this study, we evaluated parental work information recorded on the birth certificate in which the current methods of job and industry classification were used. We compared the maternal and paternal occupation recorded on the birth certificate with information obtained from telephone interviews of mothers of births with and without major malformations. We also examined the degree of misclassification as unemployed (including homemakers, students, disabled, otherwise unemployed) and the proportions of missing values on the birth certificate across parental occupations and industrial sectors of work.

## Methods

### Study population

The study population consisted of Texas participants in the National Birth Defects Prevention Study (NBDPS) who gave birth to offspring with selected congenital defects or births without major birth defects during 1997–2000. The NBDPS is a population-based, case-control study that has been ongoing since 1997 and includes study populations in 10 states [12,13]. During the study years of 1997–2000, Texas participants were mainly recruited from 105 counties in west and south-central Texas. For the purposes of the present study, birth defect cases were included if they had neural tube defects (British Pediatric Association [BPA] Classification of Disease Codes 740.000–742.090), oral clefts (BPA codes 749.000–749.220), or conotruncal heart defects (BPA codes 745.000–745.010, 745.100–745.190, 745.200–745.210, 747.215, 747.230, 747.250, 746.000–746.090 with 745.480 or 745.490 with muscular ventricular septal defects [VSDs] excluded, 746.995 with 745.480 or 745.490 with muscular VSDs excluded, 747.310, or 746.840). The Texas Birth Defects Registry staff linked the Texas NBDPS cases and controls to their respective live birth and fetal death certificates. Because this study focused on quality of parental occupational information on the birth certificate, terminations and spontaneous fetal deaths without these vital records were not included. Twenty fetal deaths (3% of the total study population including cases and controls) were not included in analyses because parental occupation and place of employment were not available in the computerized fetal death certificate files.

### Data collection

In the NBDPS, interviews are targeted for completion within 6 months of delivery with the maximum time from delivery/termination to interview of no more than 24 months [13]. A higher proportion of case-mothers have shorter gestations than control mothers due to elective terminations or fetal deaths. Therefore, to minimize recall bias, no women are interviewed until six weeks after the estimated due date (or delivery of a full-term infant). As part of the telephone interview using the NBDPS Mother Questionnaire, participants were questioned about the fathers' and their jobs and companies of employment during the period of three months prior to conception until the birth or termination of the index birth. We focused on maternal jobs held during the first trimester and paternal jobs held during the periconceptional period (three months prior through three months after the estimated date of conception). We examined paternal occupations during the first trimester as well as during preconception since some occupations might involve "take-home" exposures [14,15].

Job titles and industries on the NBDPS questionnaires were coded by the Centers for Disease Control and Prevention according to the SOC and NAICS systems [10,11]. We also used these classification systems to code maternal and paternal "usual occupation" and "type of business" in the computerized live birth certificate files. Study protocols were approved by the institutional review boards at the Texas Department of State Health Services and Texas A&M University. As part of the NBDPS protocol, oral consent to an interview was obtained from study women prior to conducting the interview.

### Data analysis

We examined whether occupational and industrial codes between the birth certificate and interview agreed for each Texas study participant. If the codes differed, we also compared job titles and industrial names and descriptions between the two sources. Some mothers and fathers had more than one job during the exposure windows of interest; jobs and industries were considered in agreement if at least one job or industry identified in the interview was in agreement with that listed on the subject's birth certificate. All assignments were made without knowledge of case or control status. In addition to examining overall agreement of the two sources by job title and place of work, percentages of agreement were examined by case/control status, maternal age at delivery, maternal education and race/ethnicity, and periconceptional folic acid use. Percentages of agreement were calculated with missing values included and excluded. The chi-square statistic or Fisher exact test (for any table with one or more cells with an expected frequency of less than 5) were used to assess the chance likelihood of observed variability [16].

For both data sources, job titles were grouped into the SOC major groups except community and social services occupations, health care practitioner and technical occupations, and construction and extraction occupations which were divided into finer categories. Industry of work was grouped by NAICS sector with the exception of wholesale and retail trade which were combined. With the interview information considered as the "gold standard", we calculated sensitivity (number of workers classified as working in a given category according to both birth certificate and interview divided by the number of workers identified as working in the respective category by interview), specificity (number of workers classified as not working in a given category according to both birth certificate and interview divided by the number of workers identified as not working in the respective category by interview), and 95% confidence intervals for each measure with statistical programs developed by Abramson and Gahlinger [16]. To assess agreement of parental occupation/industry information on the birth certificate with interview information, we also computed the kappa statistic, a measure that takes into account the probability of chance agreement [16,17]. We used the criteria proposed by Landis and Koch [18] to evaluate the strength of agreement as measured by the kappa statistic. Kappa statistics less than 0.00 were considered poor agreement; 0.00 – 0.20 slight agreement; 0.21 – 0.40 fair agreement; 0.41 – 0.60 moderate agreement; 0.61 – 0.80 substantial agreement; and 0.81 – 1.00 almost perfect agreement. The analyses of sensitivity, specificity, and kappa were restricted to occupations and industrial sectors with 10 or more workers by interview.

We also examined the distributions of missing values and misclassification as unemployed (homemaker, student, disabled, retired, or unemployed) for maternal and paternal occupations and industrial sectors. For maternal occupations and industrial sectors, "students" were combined with "homemakers" because of the small percentage (2.9%) of women misclassified as students on the birth certificate.

## Results

### Overall Agreement Between Data Sources

A total of 307 case-births with neural tube defects, conotruncal heart defects, or oral clefts and 342 control births among Texas participants of the NBDPS were linked to their live birth certificates (1997–2000 births). This sample represented 100% of the Texas 1997–2000 controls, 88% of the conotruncal heart cases, 88% of the oral cleft cases, and 58% of the neural tube defect cases. Including participants with missing information, 77% of the maternal occupations and 63% of the paternal occupations agreed between the data sources. Excluding participants with missing information, 78% of the maternal occupations and 75% of the paternal occupations agreed. Table 1 shows the overall agreement with occupation and industry between the birth certificate and interview by case-control status. Approximately 75% of case-mothers' and 79% of control-mothers' occupations were the same between the birth certificate and interview. Excluding participants with missing information had little impact on the overall percentage of agreement between data sources for maternal occupation. In contrast, 64% of the case-fathers' and 63% of the control-fathers' occupations were the same if participants with missing information were included, but 76% and 74% respectively if these subjects were excluded. For both mothers and fathers, percentage agreement regarding place of work was slightly less than that for occupation.

**Table 1:** Agreement between NBDPS Interview and Birth Certificate for Parental Occupation and Industry by Case-control Status

	Mother^a^						Father^b^					
	
	Cases			Controls			Cases			Controls		
	
	N	% Missing included	% Missing excluded	N	% Missing included	% Missing excluded	N	% Missing included	% Missing excluded	N	% Missing included	% Missing excluded
Occupation												

Agreement	226	74.6	76.9	263	78.7	79.7	184	64.1	76.0	198	62.7	74.4
Disagreement	68	22.4	23.1	67	20.1	20.3	58	20.2	24.0	68	21.5	25.6
Unknown^c^	9	3.0	-	4	1.2	-	45	15.7	-	50	15.8	-
Total	303	100.0	100.0	334	100.0	100.0	287	100.0	100.0	316	100.0	100.0

Industry												

Agreement	216	71.3	74.5	256	76.6	77.8	179	63.0	76.2	187	59.2	73.3
Disagreement	74	24.4	25.5	73	21.9	22.2	56	19.7	23.8	68	21.5	26.7
Unknown^c^	13	4.3	-	5	1.5	-	49	17.3	-	61	19.3	-
Total	303	100.0	100.0	334	100.0	100.0	284	100.0	100.0	316	100.0	100.0

^a^In the interview, 4 case mothers and 8 control mothers were missing information about occupation and industry.

^b^In the interview, 20 case fathers and 26 control fathers were missing information about occupation and 23 case fathers and 26 control fathers were missing information about industry.

^c^Unknown category includes subjects with missing information on the birth certificate.

Because cases and controls were similar with respect to agreement of the birth certificate information with interviews, we combined these groups in analyses of comparisons of agreement by selected maternal characteristics and specific occupations and industries. Table 2 shows the percentage of agreement between the two data sources for parental occupation by maternal age, education, race/ethnicity, and reported folic acid use. Overall, agreement between the two sources varied significantly (p ≤ 0.05) by maternal age (maternal and paternal occupation), education (paternal occupation), race/ethnicity (maternal occupation), and folic acid use (paternal occupation). For paternal occupations, the lowest proportions of agreement between the birth certificate and interview were observed among births to women less than 25 years of age (68%), with less than 12 years of education (63%), and of non-Hispanic Black race/ethnicity (64%). No consistent patterns for agreement were noted for maternal occupation by maternal age and education. Among the race/ethnicity groups studied, the lowest percentages of agreement between the two data sources for maternal occupation occurred among non-Hispanic Black women (55%). Although the percentages of agreement varied little with missing values of maternal occupation included or excluded, wide fluctuations in agreement were noted for paternal occupation when missing values were included or excluded, especially by maternal age, education, and race/ethnicity. These differences in agreement for paternal occupation were most marked for women less than 25 years of age, with less than 12 years of education, and of non-Hispanic Black race/ethnicity.

**Table 2:** Agreement between NBDPS Study Interview and Birth Certificate for Parental Occupation by Maternal Characteristics

	Maternal Occupation				Paternal Occupation			
	
		Agreement^a^				Agreement^a^		
				
Maternal characteristic	N (%) missing	N	% Missing included	% Missing excluded	N (%) missing	N	% Missing included	% Missing excluded
Age at delivery (in years)								

< 18	4 (6.1)	51	77.3	82.3^b^	33 (50.0)	22	33.3	66.7^c^
18 – 19	4 (5.6)	46	64.8	68.7	27 (38.0)	26	36.6	59.1
20 – 24	10 (5.8)	118	68.6	72.8	40 (23.3)	94	54.7	71.2
25 – 29	3 (1.8)	128	78.5	80.0	23 (14.1)	110	67.5	78.6
30 – 34	2 (1.7)	104	87.4	88.9	14 (11.8)	88	73.9	83.8
35 – 39	1 (2.3)	30	69.8	71.4	2 (4.7)	31	72.1	75.6
40 or older	1 (6.7)	12	80.0	85.7	2 (13.3)	11	73.3	84.6

Education (in years)								

0 – 8	2 (3.8)	42	80.8	84.0	17 (32.7)	22	42.3	62.9^d^
9 – 11	4 (3.0)	98	74.2	76.6	47 (35.6)	54	40.9	63.5
12	9 (4.1)	153	70.2	73.2	45 (20.6)	134	61.5	77.5
13 – 15	3 (1.9)	126	79.2	80.8	22 (13.8)	107	67.3	78.1
16 or more	1 (1.2)	70	86.4	87.5	4 (4.9)	64	79.0	83.1

Race/ethnicity								

Non-Hispanic White	3 (1.4)	166	76.1	77.2^c^	30 (13.8)	151	69.3	80.3
Non-Hispanic Black	2 (9.1)	11	50.0	55.0	8 (36.4)	9	40.9	64.3
Hispanic	13 (3.5)	282	76.4	79.2	90 (24.4)	202	54.7	72.4
Other	1 (3.2)	29	93.5	96.7	5 (16.1)	19	61.3	79.2

Periconceptional folic acid use^e^								

Yes	5 (2.2)	175	77.1	78.8	25 (11.0)	163	71.8	80.7
No	15 (3.6)	314	75.3	78.1	111 (26.6)	219	52.5	71.6^d^

^a ^N represents the number in each category that agreed. Missing cases are not included in chi-square statistics.

^b ^p = 0.05, Fisher's exact probability.

^c ^p < 0.05, Fisher's exact probability.

^d ^p < 0.05.

^e ^Periconceptional folic acid use included any use during the period one month prior through one month post concepton.

### Agreement by Occupational Groups

For all maternal and paternal occupational groups studied, the specificity of the birth certificate was 95% or better indicating that if a parent was not working within a specific occupational group according to the interview, the birth certificate most of the time identified the parents as not working in the given group. For maternal occupations with 10 or more workers by interview, the percent of women misclassified as unemployed on the birth certificate varied considerably across occupational groups, ranging from 0 percent for women working as health diagnosing and treating practitioners to 65 percent for women working in food preparation and serving occupations (Table 3). Among employed women according to both sources, sensitivity of the birth certificate for maternal occupation ranged from 70% for health technologists and technicians to 100% for health diagnosing and treating practitioners. For all maternal occupations studied, kappa statistics indicated either substantial or almost perfect agreement.

**Table 3:** Birth Certificate Compared with Interview for Maternal Occupation During First Trimester

		Women employed outside home according to both sources^a^					
		
Standard Occupational Classification (SOC) group	Number (%) misclassified as homemaker or student on vital record	Number by interview	Number by birth certificate^b^	Number by both sources	Sensitivity (%) (95% CI)	Specificity (%) (95% CI)	Kappa
Management	2 (10.5)	17	19	14	82.4 (59.1 – 95.3)	97.7 (94.9 – 99.1)	0.76
Education, training, & library	4 (15.4)	22	22	19	86.4 (67.2 – 96.4)	98.6 (96.2 – 99.6)	0.85
Health diagnosing & treating practitioners	0 (0.0)	14	17	14	100 (80.7 – 100)	98.6 (96.3 – 99.7)	0.90
Health technologists & technicians	2 (16.7)	10	7	7	70.0 (38.0 – 91.7)	100 (98.7 – 100)	0.82
Health care support	4 (23.5)	12	12	10	83.3 (54.9 – 97.1)	99.1 (97.0 – 99.9)	0.82
Food preparation & serving	26 (65.0)	14	11	10	71.4 (44.6 – 90.2)	99.5 (97.8 – 99.9)	0.79
Sales & related occupations	30 (44.1)	37	37	32	86.5 (72.6 – 94.9)	97.4 (94.4 – 99.1)	0.84
Office & administrative support	21 (24.7)	63	56	49	77.8 (66.3 – 86.8)	95.9 (92.0 – 98.2)	0.76
Production occupations	6 (30.0)	14	13	12	85.7 (60.3 – 97.5)	99.5 (97.8 – 99.9)	0.88

^a^Mothers who were listed as homemaker, student or unemployed are excluded in these analyses. Only occupations with 10 or more workers by interview are shown in Table.

^b^One missing occupation each for health care support, sales, and office support occupations.

Among the paternal occupational groups with 10 or more workers by interview, sensitivity of the birth certificate ranged from 35% for health care support occupations to 100% for health diagnosing and treating practitioners and for military-specific occupations (Table 4). Kappa statistics for paternal occupations ranged from substantial to almost perfect agreement with the exception of five occupations with kappa statistics in the moderate agreement range. Although few fathers were misclassified as unemployed on the birth certificate, the proportion of missing values varied considerably across occupational groups. While less than 10% of occupations were missing for fathers employed in management, business/financial operations, health diagnosing and treatment, and extraction (mining) occupations, 20% or more occupations were missing for food preparation and serving, building and grounds cleaning and maintenance, military, and construction occupations.

**Table 4:** Birth Certificate Compared with Interview for Paternal Occupation During Periconceptional Period^a^

		Fathers employed according to both sources					
		
Standard Occupational Classification (SOC) group^b^	Number (%) missing occupation on vital record	Number by interview	Number by birth certificate^c^	Number by both sources	Sensitivity (%) (95% CI)	Specificity (%) (95% CI)	Kappa
Management	4 (5.8)	65	58	41	63.1 (50.9 – 74.1)	95.8 (93.5 – 97.5)	0.62
Business/financial operations	0 (0.0)	12	11	9	75.0 (45.9 – 93.2)	99.6 (98.6 – 99.9)	0.78
Health diagnosing & treating practitioners	1 (8.3)	11	13	11	100 (76.2 – 100)	99.6 (98.6 – 99.9)	0.92
Food preparation & serving	12 (30.0)	27	11	10	37.0 (20.6 – 56.2)	99.8 (98.9 – 99.9)	0.51
Building and grounds cleaning and maintenance	6 (25.0)	18	10	8	44.4 (23.2 – 67.3)	99.6 (98.6 – 99.9)	0.56
Sales & related occupations	6 (12.8)	39	32	20	51.3 (35.8 – 66.6)	97.2 (95.4 – 98.5)	0.53
Office & administrative support	6 (13.6)	37	15	13	35.1 (21.1 – 51.4)	99.5 (98.5 – 99.9)	0.48
Farming, fishing, and forestry occupations	2 (14.3)	11	8	6	54.5 (25.9 – 81.0)	99.6 (98.6 – 99.9)	0.62
Construction	18 (22.5)	61	65	47	77.0 (65.3 – 86.3)	95.6 (93.3 – 97.3)	0.71
Extraction workers	1 (7.7)	12	8	6	50.0 (23.4 – 76.6)	99.6 (98.6 – 99.9)	0.59
Installation, maintenance, & repair	6 (16.2)	26	32	18	69.2 (49.8 – 84.6)	96.9 (94.9 – 98.2)	0.60
Production occupations	13 (18.6)	57	57	42	73.7 (61.2 – 83.9)	96.4 (94.3 – 97.9)	0.70
Transportation & material moving	12 (16.0)	59	58	41	69.5 (56.9 – 80.2)	95.9 (93.6 – 97.5)	0.70
Military-specific occupations	6 (23.1)	19	23	19	100 (85.4 – 100)	99.1 (97.9 – 99.7)	0.90

^a ^Three months before through three months postconception.

^b ^Only occupations with 10 or more workers by interview are shown in Table.

^c^One father each misclassified as not employed (student, disabled or retired, homemaker, unemployed) for food preparation and serving, sales, office support, agricultural, construction, and military occupations. Two fathers in transportation occupations and four fathers in installation/maintenance/repair occupations misclassified as unemployed.

### Agreement by Industrial Sectors

Among industrial sectors with 10 or more workers by interview, specificities of industrial sectors on the birth certificate ranged from 93.4 to 99.5%, and sensitivities ranged from 55 to 100%. Except for the administration and support sector, kappas for maternal and paternal industrial sectors fell within the substantial or almost perfect categories of agreement. Sensitivities, specificities, and kappas for industrial sectors are shown in Tables 5 and 6. Few women were missing information regarding industrial sector but were frequently misclassified as not employed on the birth certificate. This misclassification ranged from 15% for women working in the health care and social assistance sectors to 67% for women in the accommodation and food services sectors (Table 5). Paternal industrial sectors with the highest proportion of missing values included administration and support (38%), accommodation and food services (25%), and construction (24%).

**Table 5:** Birth Certificate Compared with Interview for Maternal Industrial Sectors of Work During First Trimester

		Women employed outside home according to both sources^a^					
		
North American Industry Classification System (NAICS) industrial sector	Number (%) misclassified as homemaker or student on vital record	Number by interview	Number by birth certificate^b^	Number by both sources	Sensitivity (%) (95% CI)	Specificity (%) (95% CI)	Kappa
Manufacturing	4 (22.2)	14	15	12	85.7 (60.3 – 97.5)	98.6 (96.2 – 99.6)	0.82
Wholesale & retail trade	25 (36.8)	42	40	35	83.3 (69.8 – 92.4)	97.3 (94.1 – 99.0)	0.82
Finance & insurance	4 (18.2)	17	17	16	94.1 (74.3 – 99.7)	99.5 (97.7 – 99.9)	0.94
Professional, scientific, & technical services	4 (28.6)	10	9	7	70.0 (38.0 – 91.7)	99.1 (97.0 – 99.9)	0.73
Administration & support; waste management	8 (38.1)	12	11	7	58.3 (30.2 – 82.8)	98.1 (95.6 – 99.4)	0.59
Educational services	8 (21.1)	29	27	24	82.8 (65.8 – 93.4)	98.5 (95.9 – 99.6)	0.84
Health care & social assistance	10 (15.4)	52	53	48	92.3 (82.5 – 97.5)	97.2 (93.8 – 98.9)	0.89
Accommodation & food services	31 (67.4)	15	16	13	86.7 (62.5 – 97.7)	98.6 (96.2 – 99.6)	0.83
Public administration	5 (20.8)	19	14	13	68.4 (45.5 – 86.1)	99.5 (97.7 – 99.9)	0.77

^a^Mothers who were listed as homemaker, student or unemployed are excluded in these analyses. Only industrial groups with 10 or more workers are shown in Table.

^b^One missing each for manufacturing, finance and insurance, administrative support, and educational services sectors. Three missing for health care and social assistance sectors.

**Table 6:** Birth certificate compared with interview for paternal industrial sectors of work during periconceptional period^a^

		Fathers employed according to both sources					
		
North American Industry Classification System (NAICS) industrial sector^b^	Number (%) missing industry on vital record	Number by interview	Number by birth certificate^c^	Number by both sources	Sensitivity (%) (95% CI)	Specificity (%) (95% CI)	Kappa
Agriculture, forestry, fishing & hunting	3 (14.3)	17	17	13	76.5 (52.5 – 92.0)	99.1 (97.8 – 99.7)	0.76
Mining	1 (7.1)	13	11	8	61.5 (34.1 – 84.3)	99.3 (98.2 – 99.8)	0.66
Construction	22 (24.4)	67	77	51	76.1 (64.9 – 85.2)	93.4 (90.6 – 95.6)	0.66
Manufacturing	15 (16.1)	77	71	55	71.4 (60.6 – 80.7)	95.8 (93.5 – 97.5)	0.69
Wholesale & retail trade	17 (18.9)	71	68	52	73.2 (62.1 – 82.6)	95.9 (93.6 – 97.6)	0.70
Transportation & warehousing	6 (14.6)	35	32	24	68.6 (51.9 – 82.2)	98.1 (96.5 – 99.1)	0.69
Professional, scientific, & technical services	0 (0.0)	12	14	9	75.0 (45.9 – 93.2)	98.9 (97.7 – 99.6)	0.68
Administration & support; waste management	8 (38.1)	11	14	6	54.5 (25.9 – 81.0)	98.2 (96.7 – 99.2)	0.47
Educational services	2 (9.5)	19	19	17	89.5 (69.4 – 98.2)	99.5 (98.5 – 99.9)	0.89
Health care & social assistance	2 (7.4)	22	22	19	86.4 (67.2 – 96.4)	99.3 (98.2 – 99.8)	0.86
Accommodation & food services	10 (25.0)	29	21	18	62.1 (43.7 – 78.2)	99.3 (98.1 – 99.8)	0.70
Other services (except public administration)	2 (6.3)	28	27	20	71.4 (52.9 – 85.8)	98.4 (96.8 – 99.3)	0.71
Public administration	7 (13.0)	46	47	42	91.3 (80.3 – 97.2)	98.8 (97.4 – 99.6)	0.89

^a ^Three months before through three months postconception

^b ^Only industries with 10 or more workers by interview are shown in Table.

^c^One father each misclassified as unemployed for agricultural, construction, manufacturing, accommodation/food service, and public administration sectors. Two fathers each misclassified as unemployed for wholesale/retail trade, administration/support and other services sectors. Three fathers in the health care/social assistance sector misclassified as unemployed.

## Discussion

The findings of this study indicate that parental occupation on the birth certificate may have some utility in the surveillance of occupational hazards in the workplace. Restricting comparisons to mothers who were gainfully employed according to the birth certificate and by interview, maternal occupation on these records exhibited excellent specificity and agreement (as measured by the kappa statistic) for most SOC occupational groups in which sufficient numbers of workers were available for analysis. We noted less agreement of the birth certificate with interview information regarding paternal occupation and maternal and paternal place of work than for maternal occupation. The limitations in using the birth certificate for occupational surveillance may differ for maternal and paternal occupations. In this study, mothers were frequently misclassified as homemakers or otherwise unemployed while the fathers' work information was often missing altogether. Furthermore, the problems of misclassification and missing values varied across occupational and industrial groups.

Because some parents change occupations between conception and delivery, the designation of parental occupation on the birth certificate as "usual occupation" limits the utility of using these records in the surveillance of reproductive hazards having effects early in pregnancy. Among mothers and fathers in this study population who held more than one position during the index pregnancy, the job held at delivery usually agreed between the birth certificate and interview.

### Comparison of Findings with Other Studies

As in other studies, we found overreporting of "homemaker" on the birth certificate [6,7]. This misclassification was particularly problematic among women who, according to the interview, held jobs requiring less formal education and/or technical skill such as sales and food service occupations with 44% and 65% respectively misclassified as homemakers or students on the birth certificate. In their study of New York State birth certificates, Marshall et al. [7] found that women in service occupations were the most likely to be misclassified as not employed (33%) while women in managerial/professional occupations were the least likely to be misclassified regarding employment status (6%).

The findings in this study were similar to those in New York [7] and California [8] with respect to the accuracy of maternal occupation on the birth certificate during the first trimester. Shaw et al. [8] noted that 71% of the maternal occupations on the birth certificate were similar to those obtained from an interview among case-mothers of births with severe cardiac disease and control-mothers who were residents of Santa Clara County. In the New York State study, 72% of maternal occupations and 77% of maternal industries or place of work on the birth certificate agreed with information from a mailed questionnaire. Both studies found negligible differences in agreement between cases and controls.

In the Santa Clara County study, the birth certificate was also examined for accuracy of paternal occupation for the period of three months prior to pregnancy and was noted to be comparable to interview information for approximately 80% of the records. In contrast, the birth certificate agreed for paternal occupation in only 63% of the records in the present study (with the missing cases included) in which we also included the first trimester as part of the exposure window. Part of this discrepancy might be explained by the higher proportion of unknown occupations (16%) in this study compared with the Santa Clara county study (6%). Using interview information, we examined types of occupations represented in the unknown category for birth certificates and noted a disproportionate number of occupations in food service, cleaning, construction, and the military according to the interview.

Based on finding similar misclassification of occupation on the birth certificate by case control status in previous studies, investigators [7,8] concluded that misclassification was nondifferential, and use of the birth certificate information for case-control studies would lead to risk effect estimates closer to the null (odds ratio of 1.0). In the present study, we also noted similar proportions of misclassification for maternal and paternal occupations by case-control status. Although the numbers of specific birth defects were limited in the present study, we examined the relation between several maternal and paternal occupational groups and oral clefts. Risk effect estimates obtained for selected birth defects in relation to maternal occupations as reported on the birth certificate supported the prediction of weaker associations than those obtained from a more accurate source such as by questionnaire. Using all other paternal occupations as the referent group, we noted a positive association between paternal occupations in installation, maintenance, and repair and oral clefts; this association was stronger in the birth certificate group (odds ratio [OR] 2.7, 95% confidence interval [CI] 1.3, 5.6) than by interview (OR 2.0, 95% CI 0.96, 4.4) Although misclassification of paternal occupation appeared nondifferential in this study, the proportion of specific occupational groups that were unknown varied by case-control status. For example, control-fathers were more likely than case-fathers to be missing occupation on the birth certificate if they were employed in food service, cleaning or military occupations. Since the referent group for risk estimates included fathers who worked in occupations other than the occupational group of interest, these occupational groups that were more likely to be missing on the birth certificate would be underrepresented in the referent group for the controls. This bias could result in risk estimates farther away from the null even though the misclassification of paternal occupation by case control status was equivalent. Without the interview data, this potential bias would have been missed even if the percentage of unknown paternal occupations were compared by case-control status; in this study, the percentages of missing occupations were essentially the same between the two groups (15.7% versus 15.8%). We also looked at the effect of using fathers in professional or management occupations as a referent group; in the present study, these groups had few missing data on the birth certificate and showed substantial to almost perfect agreement with the interview. With these fathers as the referent group, paternal occupations in installation, maintenance, and repair were more strongly associated with oral clefts by interview (OR 3.4, 95% CI 1.2, 9.3) than by using the birth certificate information (OR 2.6, 95% CI 0.95, 7.0).

Furthermore, the proportion of missing paternal occupations on the birth certificate varied by reported maternal folic acid use during the periconceptional period. This differential in missing information by folic acid use could introduce bias in studies of paternal occupation and neural tube defects, oral clefts, and other defects for which insufficient folic acid is a risk factor. These findings underscore the need for caution when interpreting associations of parental occupation and birth defects based on birth certificate information. Choice of referent group might lead to overestimates as well as underestimates of associations if missing occupations vary by case-control status.

Previous studies did not specifically examine the proportion of missing values on the birth certificate by parental occupation/industry. In the present study, paternal professional or managerial occupations that require more formal education were less likely to have missing information on the birth certificate than occupations requiring less education such as construction or food service occupations. Very few birth certificates were missing information regarding maternal occupation or industrial sector, but, as already discussed, mothers were frequently misclassified as homemakers.

### Limitations of the Study

This study had several limitations including use of the maternal interview as the gold standard to evaluate the quality of paternal occupational information on the birth certificate. In telephone interviews, mothers provided information about fathers' occupations and places of work. Schnitzer et al. [19] found mothers' reports of fathers' occupations to be subject to error with an exact agreement of 59% between mother's and father's reports of father's jobs in a metropolitan Atlanta population. However, mothers in the Atlanta study were interviewed 2 to 15 years after the index birth [20] in contrast to the NBDPS mothers who are interviewed 6 weeks to 2 years after the index birth. Therefore, it is likely that the mothers' reports of fathers' occupations in the NBDPS are more accurate than those of the 1968 – 1980 Atlanta birth cohort mothers from which Schnitzer et al. derived their study data, though some misclassification will still remain. Length of time from pregnancy to interview and multiple jobs during the exposure period of interest may increase errors in maternal recall of paternal occupations.

We also did not evaluate the accuracy of parental occupation in the fetal death records because this information was not available in the computerized fetal death record files in Texas. Although this exclusion amounted to only 20 cases in this study, it would be important to include fetal death records for occupational disease surveillance for several types of birth defects such as anencephaly in which fetal deaths represent a high proportion of cases.

## Conclusion

In conclusion, linkage of birth defect registry cases with their respective birth certificates and parental occupation shows some promise in surveillance of reproductive hazards in the workplace. The findings in this study also indicate that the quality of this information varies by parental status and by occupation. Misclassification of mothers as homemaker or otherwise unemployed on the birth certificate and missing information for paternal occupation may be problematic. The overall agreement between the birth certificate and maternal interview, degree of misclassification, and proportion of missing values also vary by occupations and industrial sectors. Because of these limitations, occupational surveillance findings based on the birth certificate should be followed up with studies based on parental interview and industrial hygiene assessments of workplace exposures.

## Competing interests

The author(s) declare that they have no competing interests.

## Authors' contributions

JDB conceived of and designed the study, carried out the data analyses, and wrote the first draft. LS and PHL participated in the design of the study, acquired the relevant data, assisted in the interpretation of results, and provided revisions to the manuscript. All authors read and approved the final manuscript.

## Pre-publication history

The pre-publication history for this paper can be accessed here:


